# Supplementary figures and images for: An experimental assay of the interactions of amino acids from orthologous sequences shaping a complex fitness landscape
Source: PLoS Genet. 2019 Apr 10;15(4):e1008079. doi: 10.1371/journal.pgen.1008079 (PMC6476524; doi:10.1371/journal.pgen.1008079)

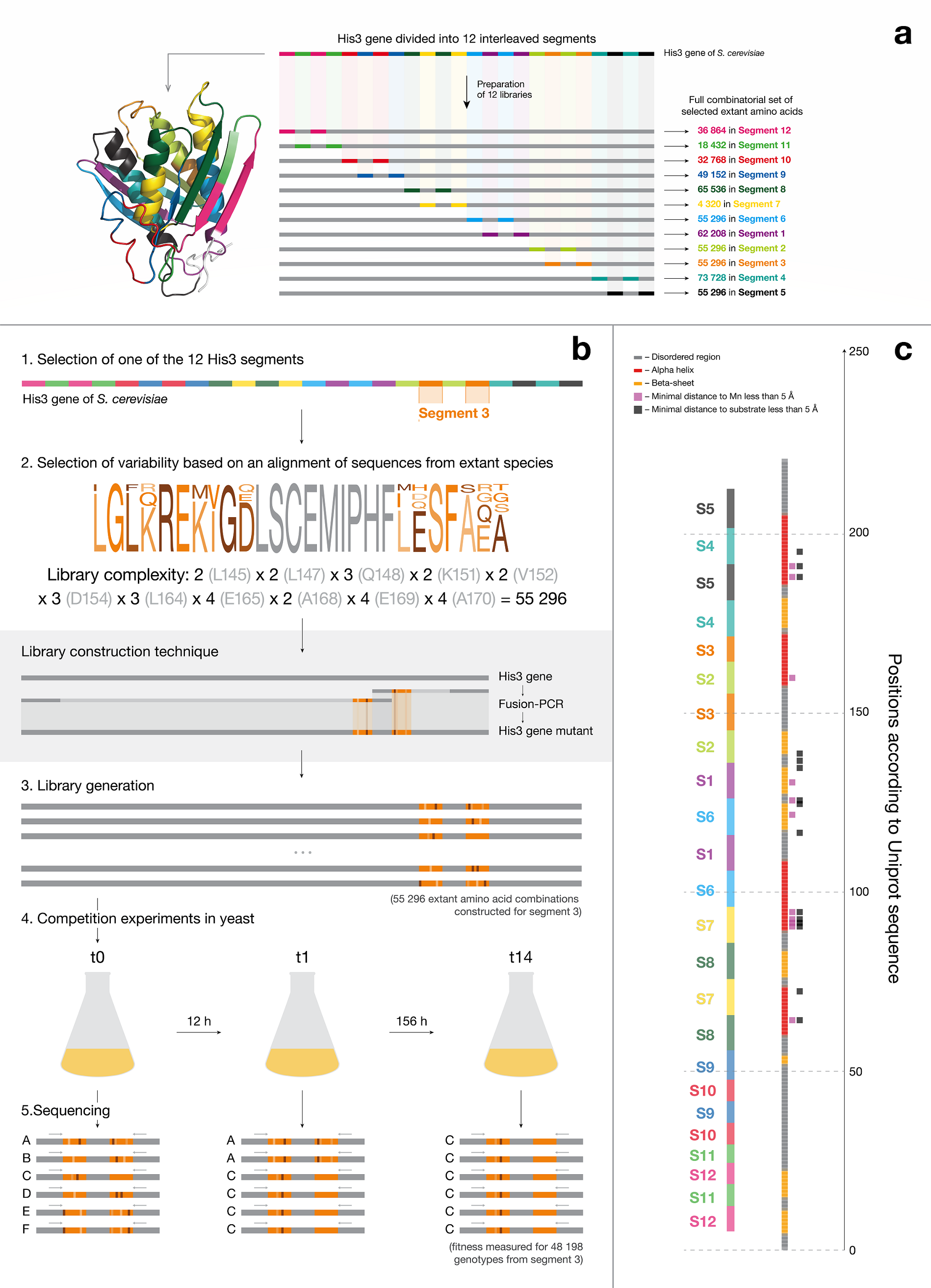

Supplement: S1 Fig — a, The sequence of the His3 protein from S. cerevisiae was separated into 12 independent segments of similar lengths, such that the full combinatorial set of extant amino acid replacements was less than 100,000 possible genotypes. These segments represented different combinations of structural elements of the His3 protein structure. b, For each of the 12 segments from His3, we selected extant amino acid states using a multiple alignment of His3 orthologues from 396 species, preferentially incorporating states from 21 yeast species, the variability is shown in segment 3 as an example. Mutant degenerate codon libraries were constructed by fusion PCR of two synthesized variable halves of each segment. These high-complexity plasmid libraries were transformed into haploid His3 knockout S. cerevisiae strain. The growth rate of yeast carrying different extant amino acid state combinations in His3 gene was measured using serial batch culture in the absence of histidine with 12 hours between ~100-fold dilutions. To estimate the fitness of yeast mutants their relative abundance was measured at three points: in the initial population before selection (t0), in the population after 12 hours of growth in the selective medium (t1), and in the final population after 168 hours of growth in the selective medium (t14). To assess the fitness of individual mutants the segments from three populations were amplified and sequenced. The relative abundance of each sequence was used as a proxy for abundance of the associated yeast mutant, which in turn determines its fitness. c, Secondary structure of His3 mapped to the segments in our experiments. (TIFF) [file pgen.1008079.s001.tiff]

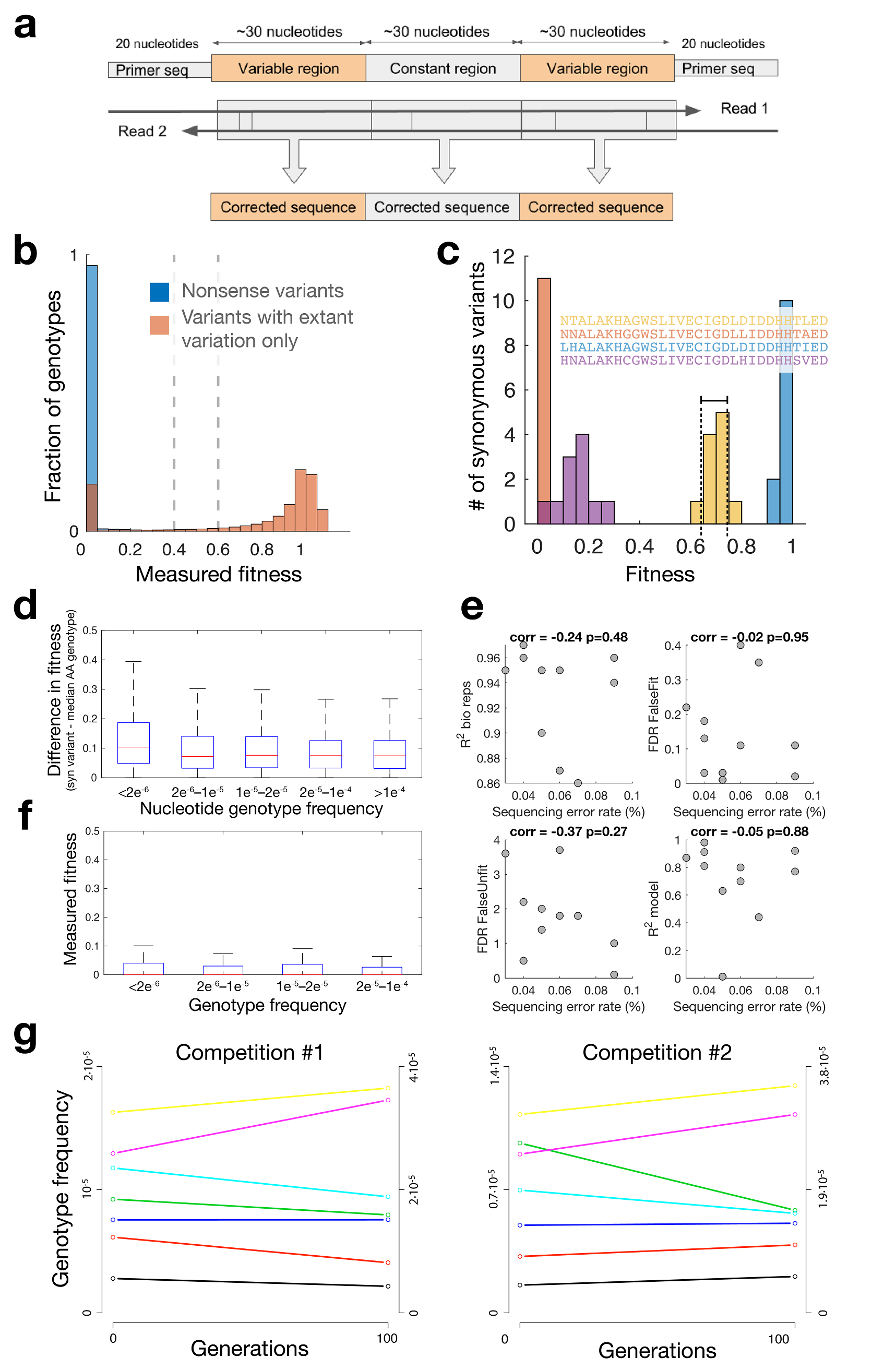

Supplement: S2 Fig — a, The pair-end reads strategy ensured that all variable regions were read twice. Mismatches between the reads were corrected to the highest quality nucleotide. The differences between the corrected sequence and the expected sequence in the constant region were used to estimate the sequencing error rate. b, Histograms of the fraction of nonsense (blue, expected fitness = 1) and extant amino acid state combinations (red). 99.63% of nonsense genotypes have a fitness > 0.4 while 23.46% of extant amino acid combinations have fitness < 0.6. c, The distribution of measured fitness of synonymous variants for four different amino-acid genotypes. d, The distribution of the difference between fitness of a nucleotide genotype with the mean fitness of all nucleotide genotypes with the corresponding amino acid sequence. We binned the measurements by the frequency of each nucleotide sequence in our data. Only amino acid genotypes with > = 10 nucleotide genotypes with measured fitness were used. Bars represent 99% of the distribution. e, Correlation of sequencing error rate in each segments, which varied 3-fold, and different measures of our data. None of the correlations were statistically significant, providing evidence that sequencing errors do not substantially contribute to our results. f, The measured fitness for all nonsense genotypes binned as a function of genotype frequency at t0. g, The measured genotype frequencies of 7 spiked-in clones that are synonymous with the wild type S. cerevisiae HIS3 gene, and therefore expected to all have nearly equal fitness, in two independent competition experiments. Note that the two y-axes in each replicate are scaled, as the absolute genotype frequencies change due to genotypes with low fitness dropping out. (TIF) [file pgen.1008079.s002.tif]

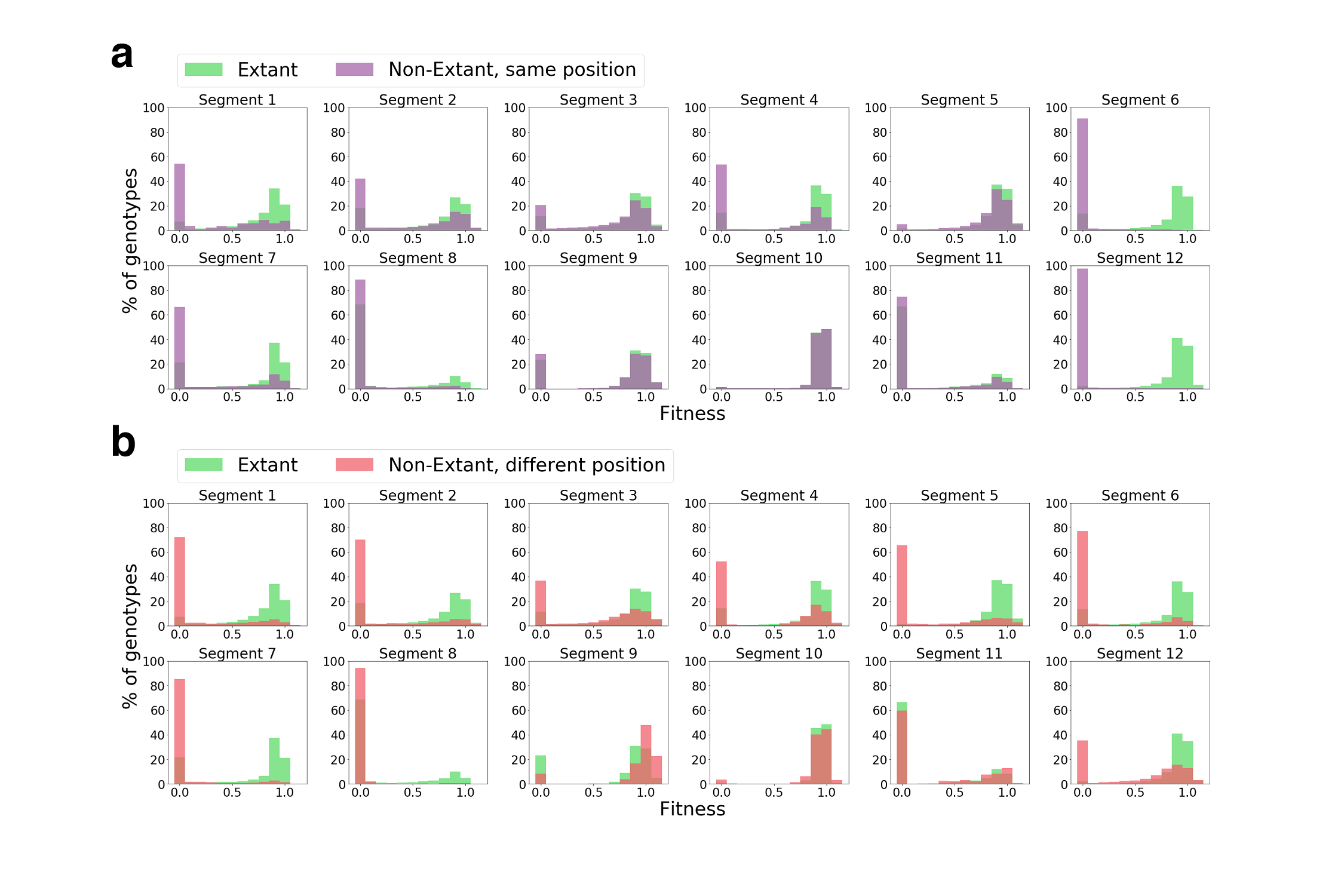

Supplement: S3 Fig — a, The fitness distribution for each segment for genotypes consisting only of extant amino acid states (green) or that contain one or more non-extant amino acid states (purple) only at positions with a replacement in the extant library. b, The fitness distribution for each segment for genotypes consisting only of extant amino acid states (green) and genotypes with mutations at other positions in that segment (red). (TIF) [file pgen.1008079.s003.tif]

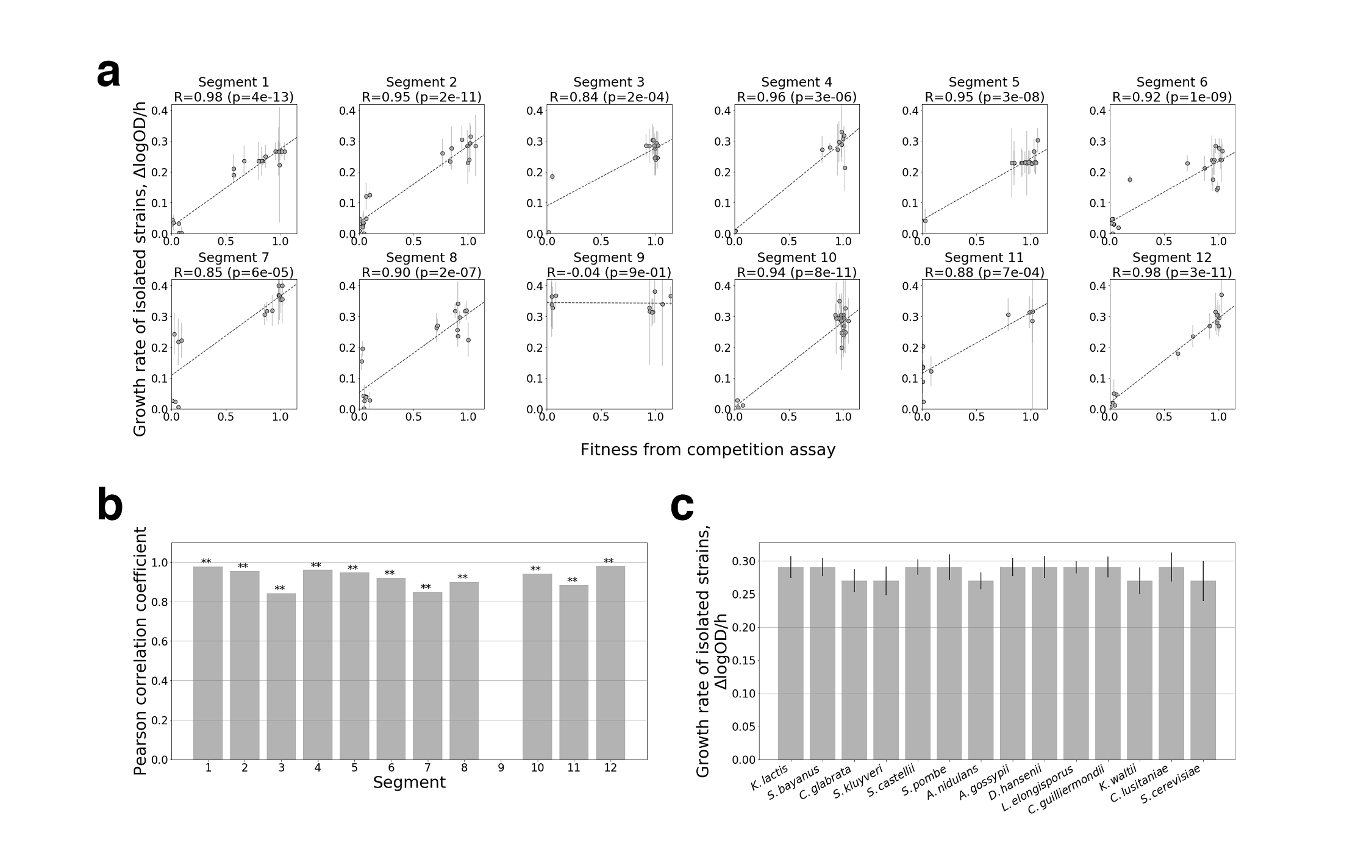

Supplement: S4 Fig — a, Comparison of fitness values from the pooled competition assay with growth rates of isolated strains as measured in a microplate reader. Error bars for growth rates show s.e.m. of 6 replicates. b, Pearson correlation coefficients between fitness values from competition and growth rates of isolated strains for each segment. ** signifies p-value < 0.005 (correlation test). c, His3p orthologues from different species complement a Δhis3 deletion in S. cerevisiae. Growth rates of transformants containing whole HIS3 orthologous genes from other yeast species. Error bars for growth rates show s.e.m. of ≥ 7 replicates. (TIF) [file pgen.1008079.s004.tif]

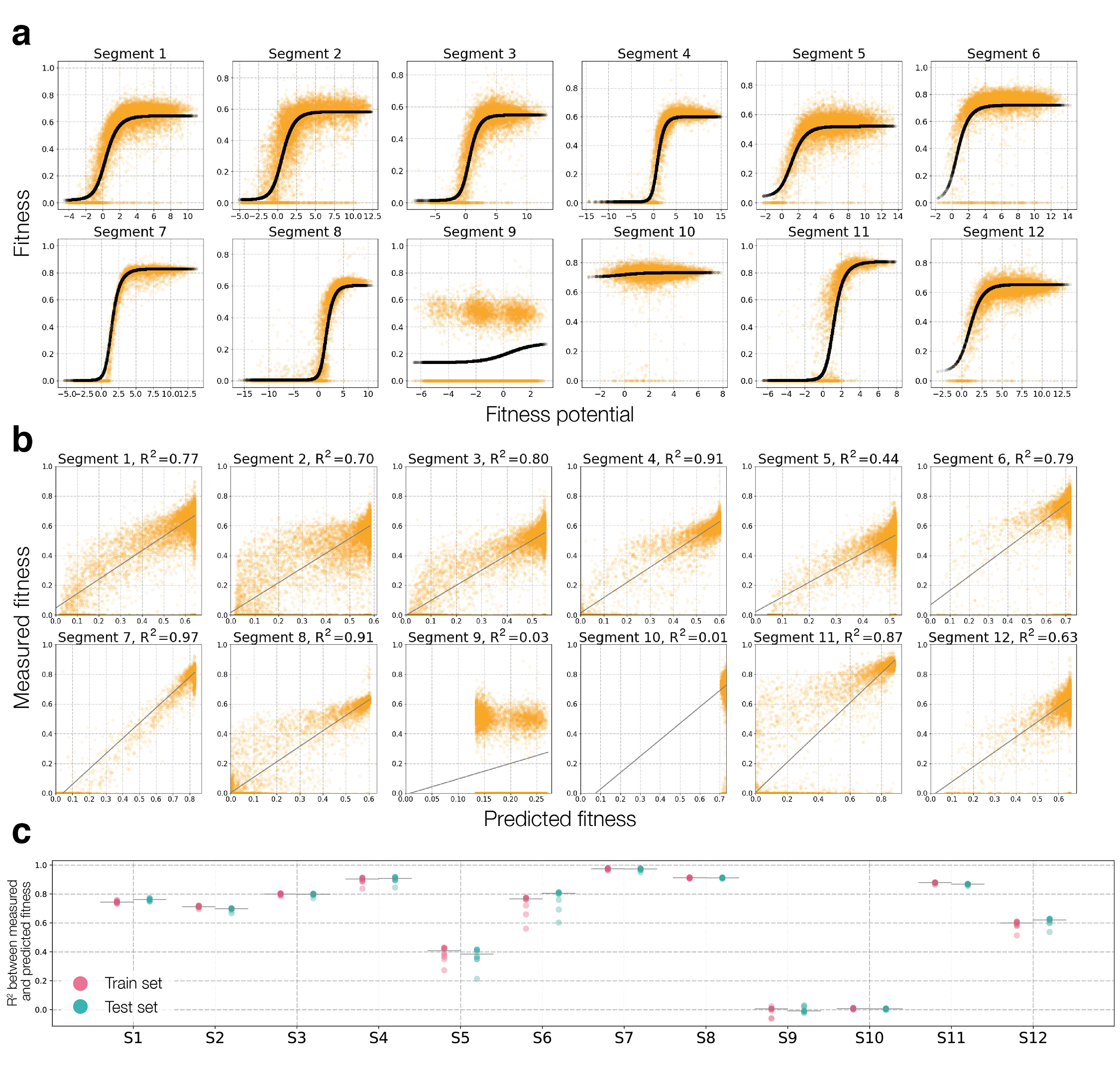

Supplement: S5 Fig — a, Fitness potential predicted by the neural network as a function of the measured fitness for all 12 segments. b, The correlation between the fitness predicted by the fitness potential and the measured fitness. c, Training and test R2 for each segment for 20-fold cross-validation. (TIF) [file pgen.1008079.s005.tif]

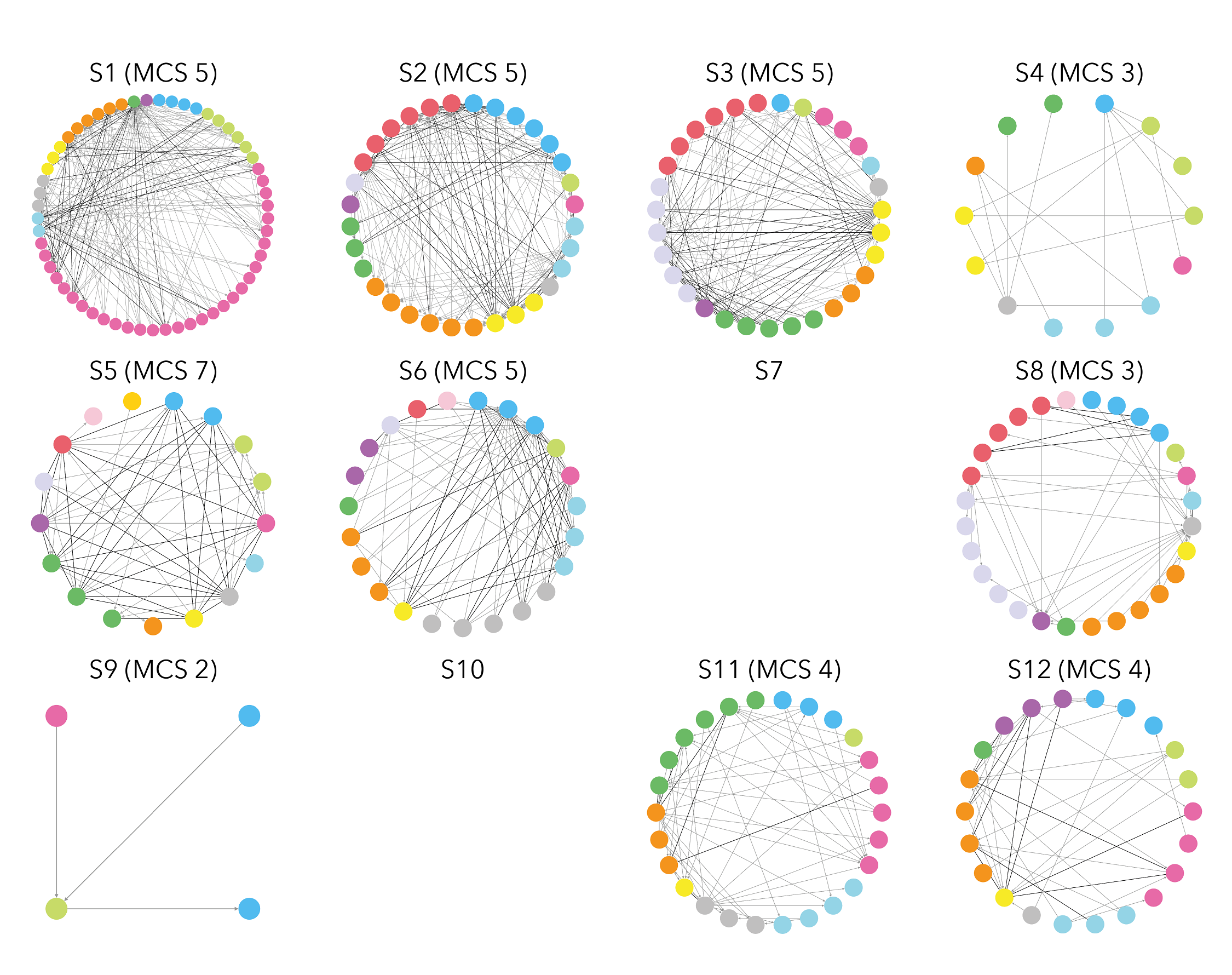

Supplement: S6 Fig — Each node represents a substitution, with multiple replacements at the same site having the same colour. Replacements under reciprocal sign epistasis are indicated by black lines while grey arrows indicate unidirectional sign epistasis. (TIF) [file pgen.1008079.s006.tif]

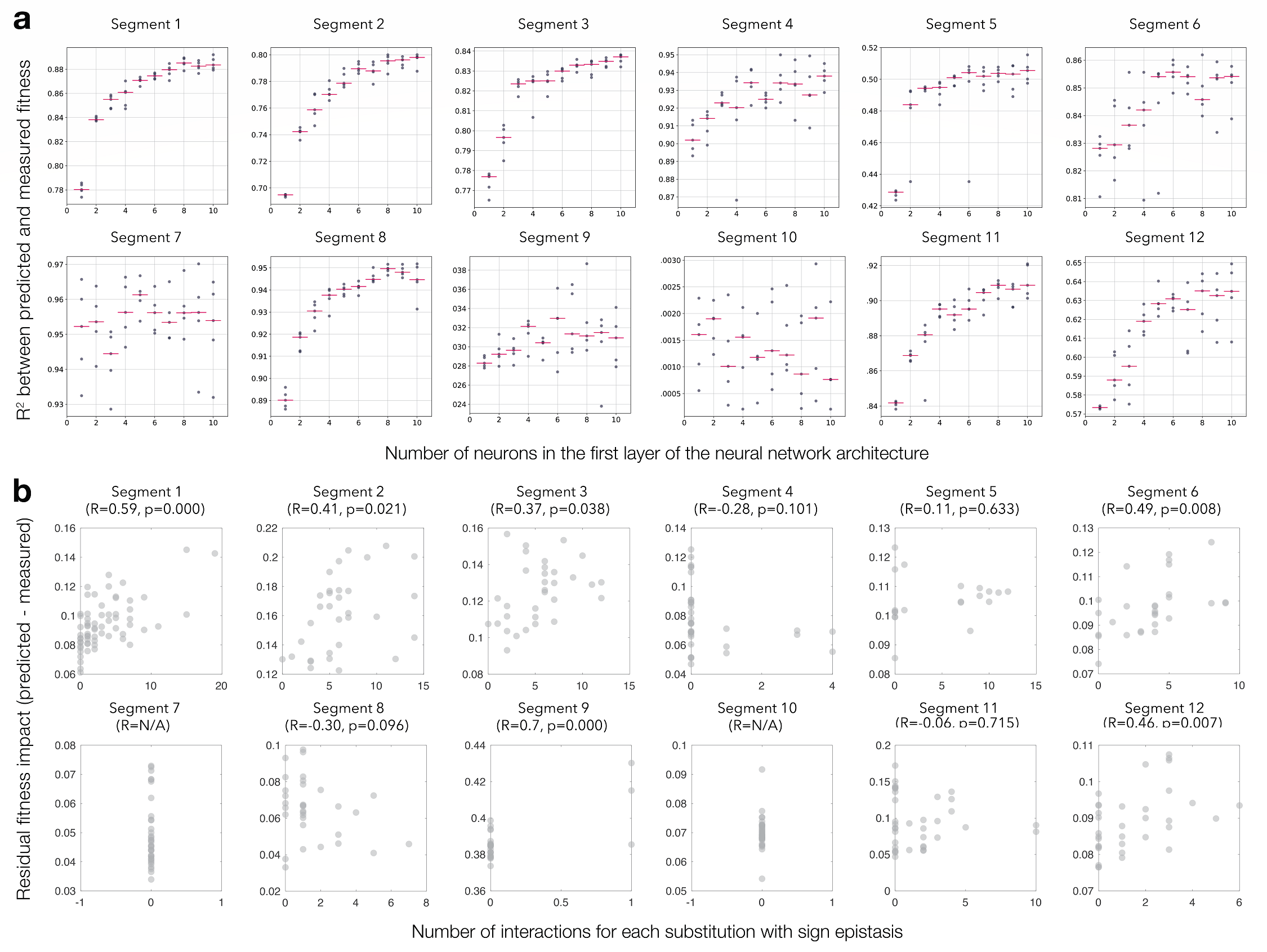

Supplement: S7 Fig — a, Increasing the number of neurons in the first layer of the neural network, which is equivalent to increasing the number of underlying fitness potentials, leads to more accurate models for segments with detected sign epistasis. Each dot corresponds to an independent optimization of model parameters. b, Number of sign epistatic interactions of certain amino acid replacements against average model prediction power for mutants including these amino acid replacements. (TIF) [file pgen.1008079.s007.tif]

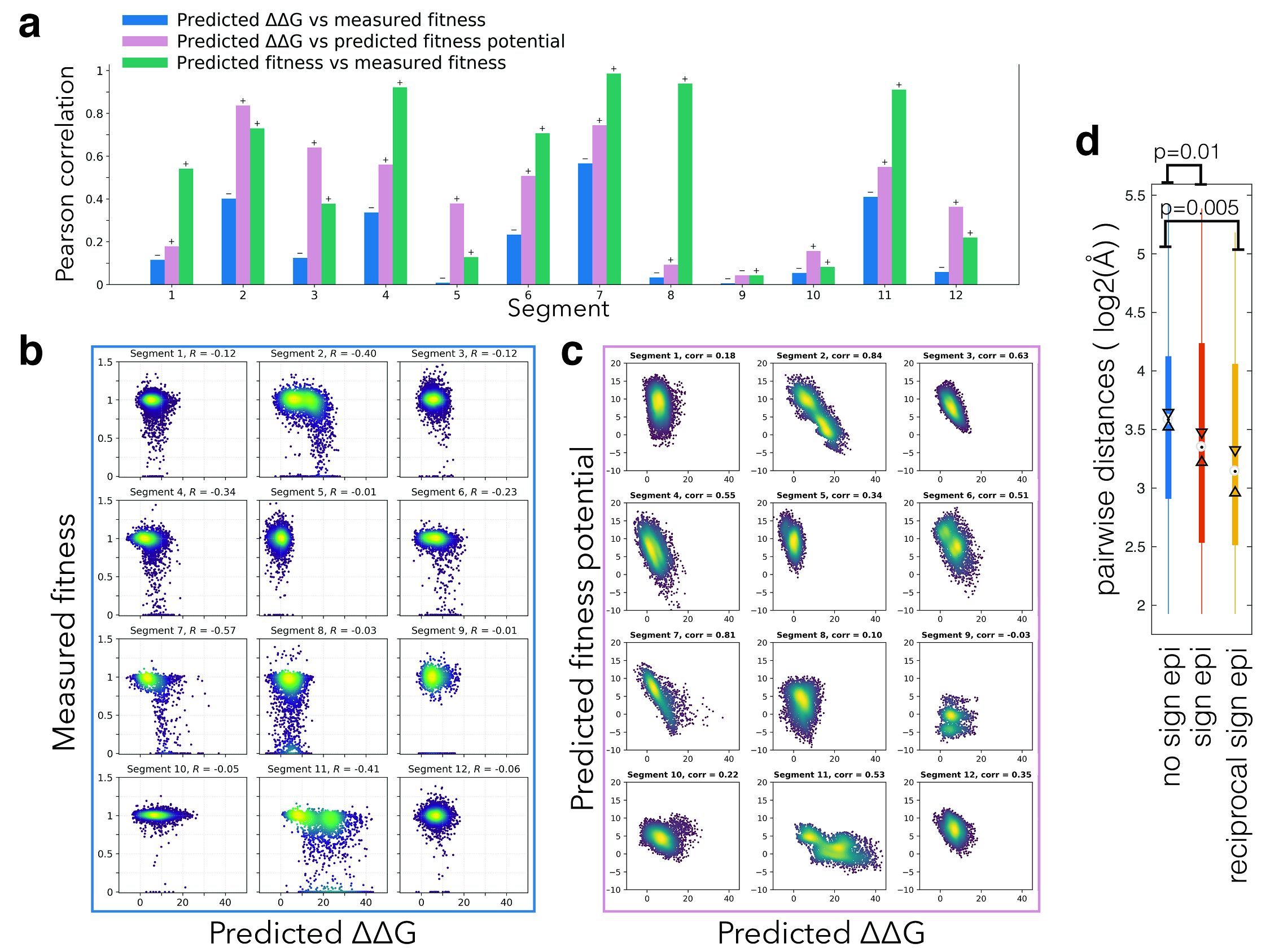

Supplement: S8 Fig — a, A comparison of correlation coefficients between predicted and measured values across segments. b,c, Correlations between the estimated impact of amino acid replacements on folding free energy (ΔΔG), fitness potential and fitness. ΔΔG correlates better with fitness potential than with fitness. d, Pairs of sites that exhibit sign (connected by a light edge in S7 Fig) and those that exhibit reciprocal sign epistasis (connected by a dark edge in S7 Fig) are closer together in the His3p structure than randomly chosen non-connected pairs of positions that exhibit sign epistasis. (TIF) [file pgen.1008079.s008.tif]

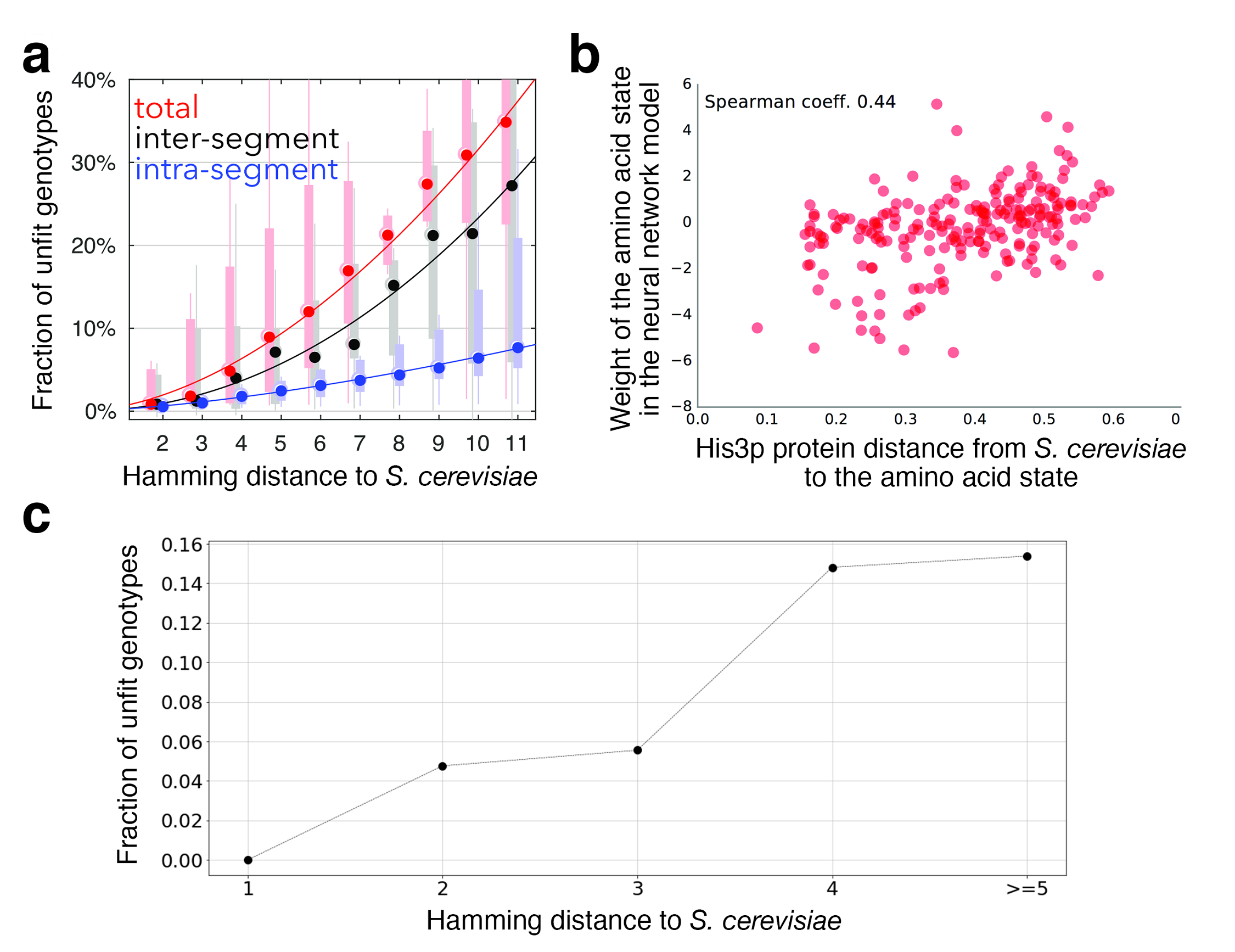

Supplement: S9 Fig — a, The fraction of unfit genotypes between S. cerevisiae and any other genotype consisting of extant amino acid states with high (blue) or any (red) fitness, and genotypes in the latter but not the former category (black) as a function of the Hamming distance between the two boundary genotypes. Points indicate median, the bars and lines indicate 50% of the genotypes and genotypes 2.7 sigmas from the mean, respectively. b, The neural network model assigns higher weights to amino acid states that first occur in His3 orthologues farther from S cerevisiae, indicating the presence of intrasegmental interactions. c, The fraction of unfit genotypes of all instances when the segment matched an extant or ancestral species as a function of Hamming distance to S. cerevisiae sequence. (TIF) [file pgen.1008079.s009.tif]

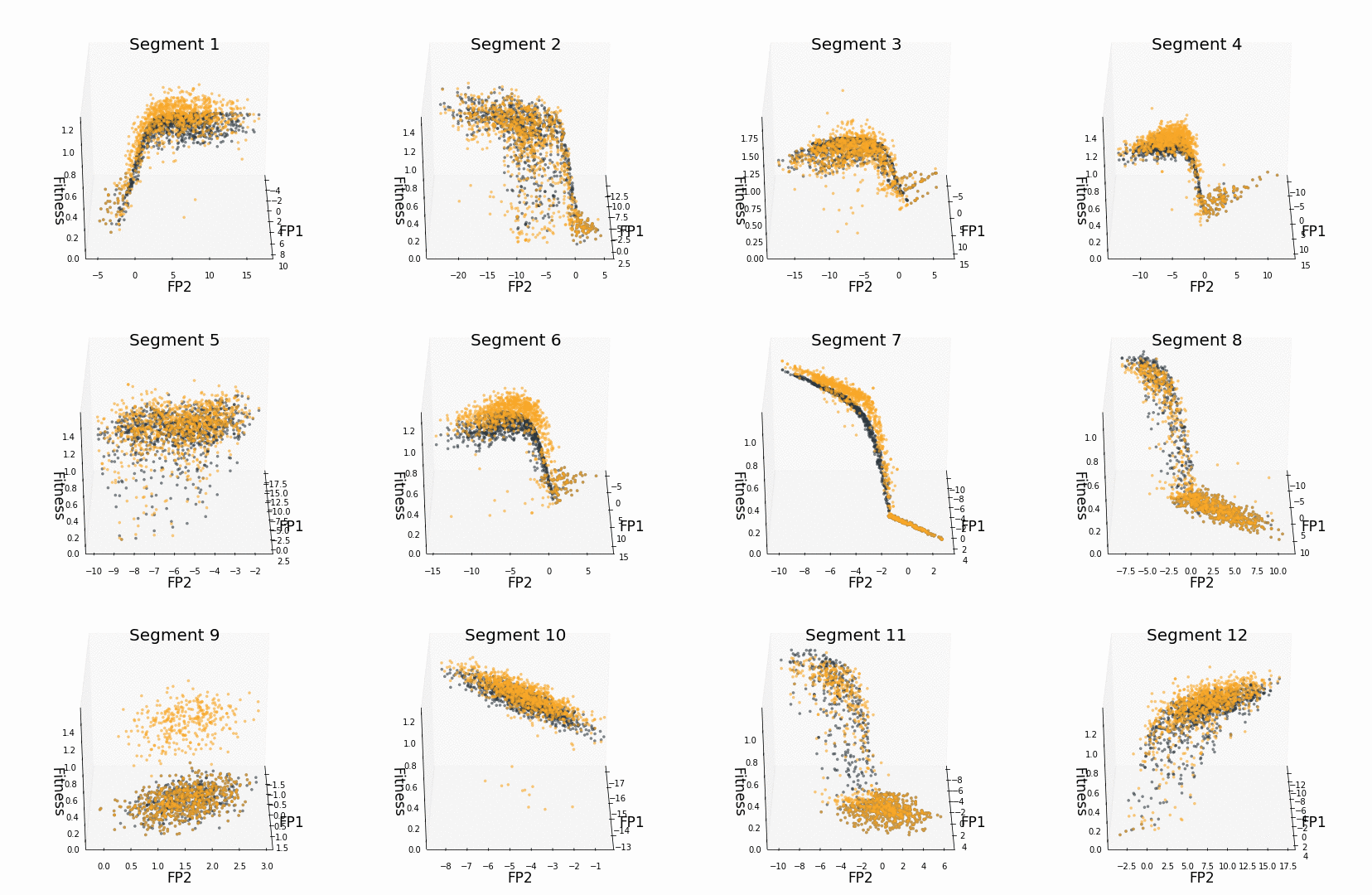

Supplement: S3 Supporting Information — Fitness as a function of two fitness potentials (black dots, measured fitness is depicted in red). (GIF) [file pgen.1008079.s012.gif]
